# Prognostic value of heart rate reserve in patients with suspected coronary artery disease undergoing stress myocardial perfusion imaging

**DOI:** 10.1007/s12350-021-02743-2

**Published:** 2021-08-03

**Authors:** Carmela Nappi, Mario Petretta, Roberta Assante, Emilia Zampella, Valeria Gaudieri, Valeria Cantoni, Roberta Green, Fabio Volpe, Leandra Piscopo, Ciro Gabriele Mainolfi, Emanuele Nicolai, Wanda Acampa, Alberto Cuocolo

**Affiliations:** 1 Department of Advanced Biomedical Sciences, University Federico II, Via Pansini 5, 80131 Naples, Italy; 2 IRCCS SDN, Naples, Italy; 3 Institute of Biostructure and Bioimaging, National Council of Research, Naples, Italy

**Keywords:** SPECT, MPI, diagnostic and prognostic application

## Abstract

**Background:**

Chronotropic incompetence is common in patients with cardiovascular disease and is associated with increased risk of adverse events. We assessed the incremental prognostic value of heart rate reserve (HRR) over stress myocardial perfusion single-photon emission computed tomography (MPS) findings in patients with suspected coronary artery disease (CAD).

**Methods:**

We studied 866 patients with suspected CAD undergoing exercise stress-MPS as part of their diagnostic program. The primary study endpoint was all-cause mortality. All patients were followed for at least 5 years. HRR was calculated as the difference between peak exercise and resting HR, divided by the difference of age-predicted maximal and resting HR and expressed as percentage.

**Results:**

During 7 years follow-up, 61 deaths occurred, with a 7% cumulative event rate. Patients experiencing death were older (*P* < .001), and had a higher prevalence of male gender (*P* < .001) and diabetes (*P* < .05). Patients with event also had lower values of HRR (65% ± 27% vs 73% ± 18%, *P* < .0001) and higher prevalence of stress-induced myocardial ischemia (25% vs 8%, *P* < .0001). Male gender, HRR and stress-induced ischemia were independent predictors of all-cause mortality (all *P* < .01). HRR improved the prognostic power of a model including clinical data and MPS findings, increasing the global χ^2^ from 66 to 82 (*P* < .005).

**Conclusions:**

Chronotropic incompetence has independent and incremental prognostic value in predicting all-cause mortality in patients with suspected CAD undergoing exercise stress-MPS. Hence, the evaluation of HRR may further improve patients’ risk stratification.

**Supplementary Information:**

The online version contains supplementary material available at 10.1007/s12350-021-02743-2.

## Introduction

The prognostic value of stress myocardial perfusion single-photon emission computed tomography (MPS) has been widely demonstrated.^1^–^3^ Mostly according to clinical status, patients referred to stress-MPS may undergo physical exercise or pharmacological test. For similar levels of coronary atherosclerotic impairment, patients able to exercise demonstrate a better outcome as compared to those undergoing pharmacological stress test due to a number of factors expressing an overall frailty.^4^

Chronotropic incompetence contributes to exercise intolerance and is associated with increased risk of adverse events.^5^–^7^ Both increased resting heart rate (HR) and chronotropic incompetence, expressed by HR reserve (HRR), are predictive of adverse outcomes, but are associated with distinct pathophysiologic processes. In particular, in patients with heart failure resting HR correlated with markers of myocardial injury and inflammation, while HRR with the neurohumoral response to exercise stress.^5^ The prognostic value of HRR beyond MPS findings has been previously reported in patients undergoing pharmacological stress test^8^; however, the added value of HRR in patients submitted to exercise stress has not been investigated. Therefore, the aim of the present investigation was to retrospectively assess the predictive value of HRR over other clinical variables and imaging findings for predicting overall mortality in patients with suspected coronary artery disease (CAD) undergoing exercise stress-MPS.

## Materials and Methods

### Study Population

The study population consisted of 3,902 consecutive patients referred to the University of Naples Federico II for suspected CAD from May 2002 and January 2014 to perform MPS after treadmill exercise as part of their diagnostic program. As part of the baseline examination clinical teams collected information on traditional cardiovascular risk factors including age, sex, hypertension, diabetes, hypercholesterolemia, smoking, family history of CAD, chest pain symptoms. Patients (n = 2,706) with previously diagnosed CAD, history of myocardial infarction (chest pain or equivalent symptom complex, positive cardiac biomarkers, or typical electrocardiographic changes), of percutaneous coronary intervention, or of coronary artery bypass grafting^9^ were excluded as well patients with severe valvular or congenital heart disease, patients with pace-maker, and significant comorbidity reducing life expectancy to < 12 months (i.e. cancer, end-stage renal disease, severe obstructive pulmonary disease). Also 298 patients undergoing early revascularization procedures (< 90 days post stress MPS) were excluded, leaving 898 patients eligible for the study. Patients were considered as having diabetes if they were receiving treatment with oral hypoglycemic drugs or insulin. A family history of premature CAD was defined as a diagnosis of CAD in a first degree relative prior to or at 55 years of age. Hypertension was defined as a blood pressure > 140/90 mmHg or use of antihypertensive medication. Hyperlipidemia was defined as total cholesterol level > 6.2 mmol·L^−1^ or treatment with cholesterol lowering medication. Smoking history was defined as prior or current tobacco use.^9^ From the answer to three questions regarding chest pain (location, precipitants, and relief with rest or nitroglycerin), we identified four chest pain categories (asymptomatic, nonanginal chest pain, atypical and typical angina).^10^ We also identified which patients complained of dyspnea only.^11^ The outcome of interest was all-cause mortality. The Review Committee of our institution approved the study and all patients gave informed consent.

### Stress MPS

Patients underwent stress-optional rest ^99m^Tc-sestamibi gated MPS by physical exercise stress test, according to the recommendations of the European Association of Nuclear Medicine^12^ as previously described.^13^ In all patients, beta-blocking medications and calcium antagonists were withhold for 48 hours and long-acting nitrates for 12 hours before testing. Symptom-limited treadmill standardized protocols were performed, with monitoring of HR and rhythm, blood pressure, and electrocardiography (ECG). Test endpoints were achievement of 85% maximal predicted HR, horizontal or down sloping ST-segment depression > 2 mm, ST-segment elevation > 1 mm, moderate to severe angina, systolic blood pressure decrease > 20 mmHg, blood pressure > 230/120 mmHg, dizziness, or clinically important cardiac arrhythmia.

At peak exercise patients were intravenously injected with standard dose of ^99m^Tc-sestamibi (8 to 10 mCi for stress and 32 to 40 mCi for rest).^14^ Patients continued the exercise for additional 60 seconds after tracer injection. HR, blood pressure, and 12-lead ECG data were recorded at rest, at the end of each stress stage, at peak stress and in the delay phases at rest. Chronotropic response was evaluated by HRR, calculated as the difference between peak exercise and resting HR, divided by the difference of age-predicted maximal HR and resting HR and expressed as percentage.^5^,^7^ Age-predicted maximal HR was calculated by (220 − age).^7^ We also calculated the peak/rest HR ratio.^15^ Resting HR was derived from the supine resting recording prior to exercise. Maximal degree of ST-segment changes at 80 ms after the J-point of the ECG was measured and assessed as horizontal, down sloping or up sloping. Imaging was started 30 minutes after tracer injection using a dual-head rotating gamma camera (E.CAM, Siemens Medical Systems, Hoffman Estates, IL, USA) equipped with a low-energy, high-resolution collimator and connected with a dedicated computer system. No attenuation or scatter correction was used. An automated software program (e-soft, 2.5, QGS/QPS, Cedars-Sinai Medical Center, Los Angeles, CA) was used to calculate left ventricular (LV) ejection fraction and the scores incorporating both the extent and severity of perfusion defects, using standardized segmentation of 17 myocardial regions.^14^,^16^ Each myocardial segment was scored from normal (score = 0) to absent perfusion (score = 4). The summed stress score, representing the total myocardium abnormal (i.e., necrotic and ischemic tissue), was obtained by adding the scores of the 17 segments of the stress images. A similar procedure was applied to the resting images to calculate the summed rest score, a measure of infarct size and severity. The summed difference score (SDS), an index of ischemic burden expressing the difference between the stress and rest scores, was converted in percent of total myocardium dividing the SDS value by 68, the maximum possible value of the 17 segments images approach. A SDS ≥ 5% of myocardium was considered an ischemic response.^4^

### Follow-up

For the 898 patients eligible for the study, alive or dead status at follow-up was ascertained by a phone call to all patients and/or general practitioners or cardiologists and by review of hospital or physicians’ records by individuals blinded to the patient’s test results. Only the occurrence of all-cause mortality was noted. The date of the last examination or consultation was used to determine follow-up. Thirty-two patients (3.6%) were lost at follow-up. All the remaining 866 patients were followed for at least 5 years and follow-up was censored at 7 years.

### Statistical Analysis

Continuous variables were expressed as mean ± standard deviation and categorical data as percentage. Differences between groups were analyzed by unpaired *t* test and χ^2^ analysis as appropriate. Two-sided *P* values < .05 were considered statistically significant. The end-point was the occurrence of all-cause mortality. Patients were grouped on quartiles of HRR and HRR was also dichotomized using the cut-off with the best trade-off between sensitivity and specificity calculated according to the Youden index.^17^ Logistic regression analysis was performed to identify the clinical and imaging predictors of a poor HRR. Event-free survival curves were obtained by the Kaplan-Meier method and compared with the log-rank test. Annualized event rates (AER), expressed as % person-years, were calculated as the cumulative number of events divided by person-time, i.e., the sum of each individual follow-up period; the Poisson regression was used to assess differences in AER among groups and to calculate the incidence rate ratio.^18^ Hazard ratios with 95% confidence intervals (CI) were calculated by univariable and multivariable Cox regression analysis. Deviance residuals were plotted against continuous variables to check the assumption of log-linear relationship with hazard. We also tried quadratic functions to represent non-linear associations. An a priori criteria for keeping the quadratic term was a significant improvement of the models by the likelihood ratio statistics. HRR-squared (*P* = .14), age-squared (*P* = .93), and METS-squared (*P* = .32) did not improved the model and were not further considered. The proportional hazard assumption was assessed by visual inspection of the log[− log(survival function)] for categorical variables and with statistical tests based on Schoenfeld residuals for both categorical and continuous variables. The proportional hazard assumption as well as the linearity of log-hazard were not rejected for any of the variables included in the Cox model. The variable considered for univariable analysis were clinical and hemodynamic data and imaging findings. Variables showing a *P* value < .05 at univariable analysis were considered for multivariable analysis. We also checked for plausible statistically significant interactions based on the likelihood ratio test. This statistic was also utilized to assess the incremental value of different models considering variables in hierarchical order (clinical data alone; clinical data and SDS; clinical data, SDS and HRR). Statistical analysis was performed with Stata 15.1 software (Stata Corp, College Station, TX).

## Results

### Patient Characteristics and Outcome

**Figure 1:**
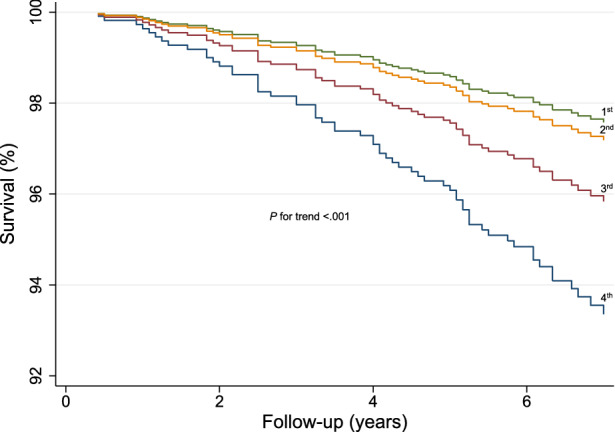
Risk-adjusted survival curves for patients across HRR quartile. On the basis of the multivariable model including age, gender, diabetes, and METS, a significant difference in survival as a function of HRR quartile was observed

The final study population included 866 patients. Baseline characteristics of these patients are presented in Table 1. For patients nonexperiencing event (n = 805), the median length of follow-up was 6.5 years (range 5-7). During follow-up, 61 deaths occurred (7% cumulative event rate) with a crude AER of 1.11% (95% CI 0.87-1.43). Characteristic of patients grouped on quartiles of HRR are reported in Table 2. There was a significant difference (*P* for trend < .001) in risk-adjusted survival curves for patients across quartiles of HRR (Figure 1).

**Table 1:** Clinical characteristics and imaging findings in 866 patients with suspected CAD undergoing exercise stress-MPS

Age (years)	58 ± 11
Male gender, n (%)	360 (42)
Diabetes, n (%)	210 (24)
Hypertension, n (%)	572 (66)
Hypercholesterolemia, n (%)	403 (47)
Current smoker, n (%)	231 (27)
Family history of CAD, n (%)	289 (33)
Chest pain symptoms	
Asymptomatic, n (%)	545 (63)
Non-anginal chest pain, n (%)	118 (14)
Atypical angina, n (%)	59 (7)
Typical angina, n (%)	144 (16)
Dyspnea, n (%)	84 (10)
Heart rate (bpm)	77 ± 14
Systolic BP (mmHg)	130 ± 17
Diastolic BP (mmHg)	81 ± 10
Metabolic equivalents	10 ± 3
HRR (%)	73 ± 19
SDS ≥ 5% (%)	76 (9)

Values are expressed as mean value ± standard deviation or as number (percentage) of subjects.

*CAD*, coronary artery disease; *BP*, blood pressure; *HRR*, heart rate reserve; *SDS*, summed difference score.

**Table 2:** Clinical characteristics and imaging findings in patients grouped on quartiles of HRR

	1 Quartile (n = 217)	2 Quartile (n = 217)	3 Quartile (n = 216)	4 Quartile (n = 216)
Age (years)	59 ± 10	58 ± 11	56 ± 11	61 ± 11
Male gender, n (%)	115 (53)	108 (50)	148 (68)	135 (63)
Diabetes, n (%)	64	52	44	50
Hypertension, n (%)	150	143	139	140
Hypercholesterolemia, n (%)	103	98	101	101
Current smoker, n (%)	57	70	50	54
Family history of CAD, n (%)	67	67	82	73
Chest pain symptoms				
Asymptomatic, n (%)	131 (60)	131 (60)	137 (63)	146 (68)
Non-anginal chest pain, n (%)	41 (19)	31 (14)	23 (11)	23 (11)
Atypical angina, n (%)	7 (3)	14 (6)	21 (10)	17 (8)
Typical angina, n (%)	38 (18)	41 (19)	35 (16)	30 (14)
Dyspnea, n (%)	15 (7)	21 (10)	26 (12)	22 (10)
Heart rate (bpm)	73 ± 13	78 ± 13	75 ± 12	80 ± 16
Systolic BP (mmHg)	131 ± 19	128 ± 18	129 ± 16	132 ± 16
Diastolic BP (mmHg)	81 ± 10	80 ± 11	81 ± 10	83 ± 8
Metabolic equivalents	9.6 ± 3.3	9.9 ± 2.8	10.8 ± 2.9	9.9 ± 3.3
HRR (%)	50 ± 10	69 ± 5	77 ± 6	94 ± 8
SDS ≥ 5% (%)	22 (10)	23 (11)	15 (7)	16 (7)

Values are expressed as mean value ± standard deviation or as number (percentage) of subjects

*CAD*, coronary artery disease; *BP*, blood pressure; *HRR*, heart rate reserve; *SDS*, summed difference score.

**Figure 2:**
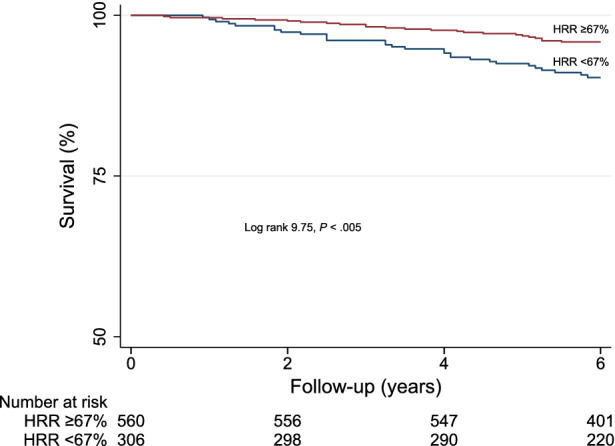
Survival curves by Kaplan-Meier according to heart rate reserve (HRR) value

**Figure 3:**
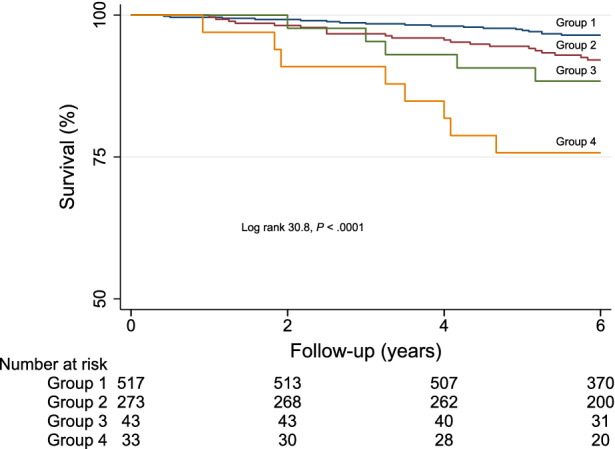
Event-free survival curves by Kaplan-Meier in patients grouped according to heart rate reserve (HRR) and summed difference score (SDS) cut-offs. Group 1: HRR ≥ 67% and SDS < 5%; group 2: HRR < 67% and SDS < 5%; group 3: HRR ≥ 67% and SDS ≥ 5%; group 4: HRR < 67% and SDS ≥ 5%

**Figure 4:**
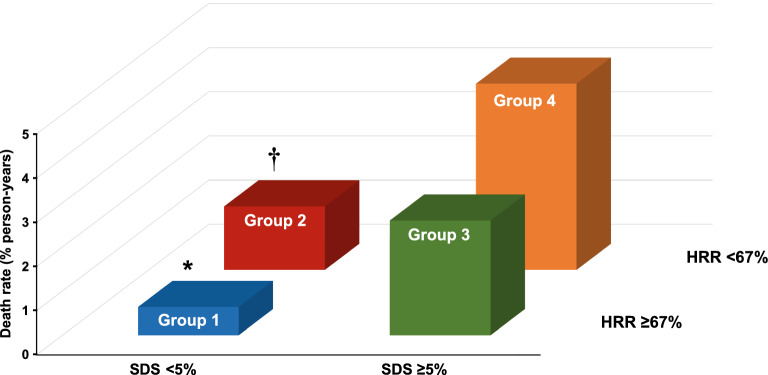
Annualized event rates in patients grouped according to heart rate reserve (HRR) and summed difference score (SDS) cut-offs. *P < .01 group 1 vs the other three groups; ^†^*P* < .01 group 2 vs group 4. The annualized event rates (% person-years) were 0.74 in group 1, 1.44 in group 2, 2.61 in group 3, and 4.22 in group 4

Based on the Youden index, a HRR of 67% provided the best trade-off between sensitivity and specificity for predicting mortality. At multivariable logistic regression analysis, among clinical variables female gender (odds ratio 1.8 age, 95% CI 1.3-2.4, *P* < .001), diabetes mellitus (odds ratio 1.4, 95% CI 1.1-1.9, *P* = .035), and METS (odds ratio 0.95, 95% CI .90-.99, *P* = .031) were independent predictors of HRR < 67%, while MPS imaging variables did not. The AER was 1.72 (95% CI 1.22-2.41) in the 306 patients with HRR < 67% and 0.79 (95% CI 0.54-1.14) in the 560 with HRR ≥67%, with an incidence rate ratio of 2.18 (95% CI 1.32-3.61; *P* < .005). Accordingly, event-free survival was significantly lower (*P* < .005) in patients with HRR < 67% compared to those with HRR ≥ 67% (Figure 2). Event-free survival analysis was also performed categorizing the patients in four groups according to HHR and SDS cut-offs (group 1: HRR ≥67% and SDS <5%; group 2: HRR < 67% and SDS < 5%; group 3: HRR ≥ 67% and SDS ≥ 5%; group 4: HRR < 67% and SDS ≥ 5%). There was a significant trend in survival function across the four groups (χ^2^ 29.6, *P* < .0001), the worst outcome being detectable in patients of group 4 (Figure 3). The annualized event rates for each of the four groups are reported in Figure 4.

### Cox Regression Analysis

**Figure 5:**
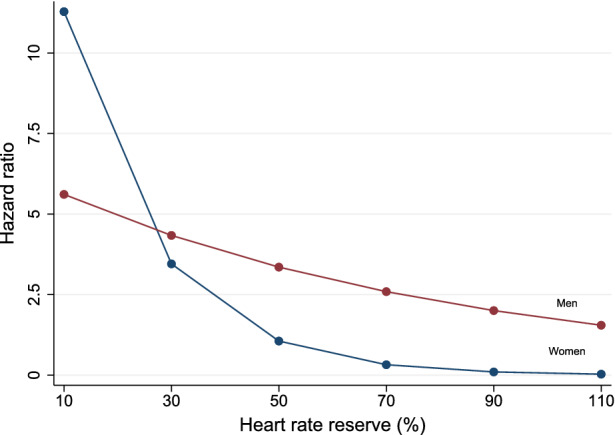
Estimated adjusted hazard ratios for heart rate reserve (HRR) in relation to time to death by gender, considering the interaction between HRR and gender

**Figure 6:**
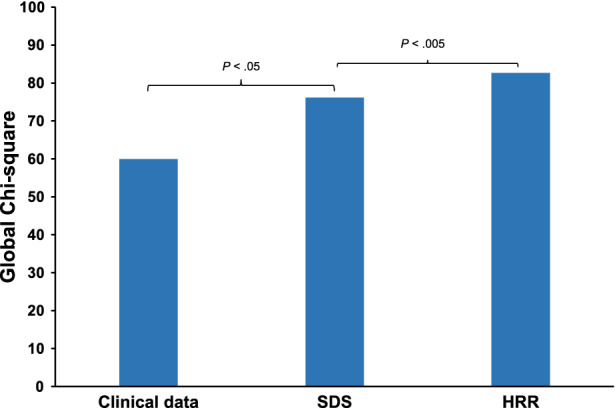
Bar graph illustrating the incremental prognostic value of heart rate reserve (HRR) over clinical data and summed difference score (SDS) for identifying patients at risk of event. The addition of HRR significantly improved the power of the model increasing the global χ^2^ value from 76.2 to 82.7

Results of univariable and multivariable Cox regression analyses are reported in Table 3. Univariable predictors of events included age, male gender, diabetes mellitus, HRR and SDS. When multivariable analysis was performed using HRR as continuous variable, age, metabolic equivalents (METS), HRR, and SDS ≥ 5%, were independent predictors of mortality. A significant interaction between gender and HRR was also found (Figure 5). When multivariable analysis was performed using HRR ≥ 67% as dichotomous variable, independent predictors of death were age, gender, METS, HRR ≥ 67%, and SDS ≥ 5%. In particular, the hazard of death was 60% lower in patients with HRR ≥ 67% compared to those with HRR below this cut-off value. No interaction was detectable between gender and HRR ≥ 67% and, therefore, this interaction was not considered in the model. HRR also improved the prognostic power of a model including clinical data and MPS findings in the prediction of all-cause mortality (Figure 6), increasing the global χ^2^ from 66 to 82 (*P* < .005).

**Table 3:** Univariable and multivariable Cox regression analyses for mortality

	Univariable analysis		Multivariable analysis			
			Model 1		Model 2	
	Hazard ratio (95% CI)	*P* value	Hazard ratio (95% CI)	*P* value	Hazard ratio (CI 95%)	*P* value
Age	1.1 (1.07-1.14)	< .0001	1.08 (1.04-1.12)	< .0001	1.07 (1.04-1.11)	< .0001
Gender^a^	4.93 (2.34-10.4)	< .0001	0.31 (0.03-3.16)	.32	5.18 (2.41-11.2)	< .001
Diabetes	1.85 (1.1-3.12)	< .05	1.57 (0.92-2.67)	.09	1.46 (0.86-2.47)	.16
Hypertension	1.28 (0.74-2.22)	.38				
Hypercholesterolemia	0.61 (0.36-1.03)	.06				
Current smoker	1.39 (0.81-2.36)	.23				
Family history of CAD	0.62 (0.34-1.12)	.11				
Chest pain symptoms^b^		.22				
Non-anginal chest pain	0.45 (0.05-3.83)					
Atypical angina	2.1 (0.74-5.95)					
Typical angina	2.02 (0.8-5.11)					
Dyspnea	1.77 (0.87-3.6)	.11				
Heart rate	0.99 (0.97-1.0)	.16				
Systolic BP	1.01 (1.0-1.03)	.14				
Diastolic BP	0.99 (0.97-1.01)	.38				
Metabolic equivalents	0.82 (0.75-0.90)	< .001	0.85 (0.77-0.94)	< .005	0.85 (0.77-0.94)	< .005
HRR^c^	0.99 (0.97-1.0)	< .05	0.94 (0.91-0.98)	< .005		
Gender × HRR			1.04 (1.01-1.09)	< .05		
HRR ≥ 67%	0.46 (0.28-0.76)	< .005			0.40 (0.24-0.67)	< .001
SDS ≥ 5%	3.6 (2.01-6.46)	< .0001	2.11 (1.16-3.80)	< .05	2.21 (1.22-3.99)	< .01

*Model 1*, multivariable Cox model including continuous HRR;* Model 2*, multivariable Cox model including dichotomous HRR;* CI*, confidence interval; *CAD*, coronary artery disease; *BP*, blood pressure; *HRR*, heart rate reserve; *HRR*, heart rate reserve; *SDS*, summed difference score.

^a^Considering female as the reference

^b^Considering asymptomatic patients as the reference

^c^For one unit increase

### Age and Gender Differences in HRR Related to Mortality

**Figure 7:**
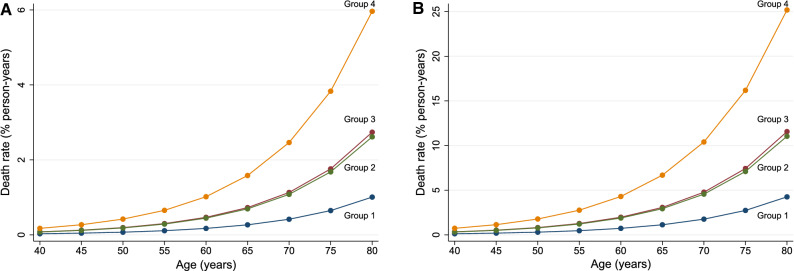
Age-adjusted annualized event rate in patients grouped according to heart rate reserve (HRR) and summed difference score (SDS) cut-offs. Panel **A** female and panel **B** male. Group 1: HRR ≥ 67% and SDS < 5%; group 2: HRR < 67% and SDS < 5%; group 3: HRR ≥ 67% and SDS ≥ 5%; group 4: HRR < 67% and SDS ≥ 5%

HRR values stratified by age and gender are reported in Table 4. The age-adjusted AER in patients grouped according to HRR and SDS cut-offs are illustrated separately for women and men in Figure 7. As shown, for each of the four groups the AER were higher in men as compared to women and progressively increased with age in both sexes.

**Table 4:** Heart rate reserve values stratified by age and gender

	Alive	Dead
Women	71 ± 18	51 ± 15
Men	74 ± 18	70 ± 28
< 60 years	71 ± 15	55 ± 18
≥ 60 years	75 ± 21	70 ± 28

### Predictive Power of Peak/Rest HR Ratio

In our study population, peak/rest HR ratio values were moderately correlated to HRR values (*r* = .40, *P* < .05). HR ratio was a significant predictor of mortality at univariable analysis (hazard ratio 0.38; 95% CI 0.16-0.92, *P* < .05), but not in a multivariable model including age, gender, diabetes, METS, and SDS ≥ 5% (adjusted hazard ratio 0.76; 95% CI 0.32-1.78, *P* = .53). When peak/rest HR ratio and HRR were tested in the same multivariable model, including age, gender, diabetes, METS, and SDS ≥5%, HRR was significant independent predictor (adjusted hazard ratio 0.98; 95% CI 0.96-0.99, *P* < .01), but HR ratio was not (adjusted hazard ratio 1.53; 95% CI 0.56-4.20, *P* = .41). The results did not change considering possible interaction with gender for both peak/rest HR ratio and HRR.

### Predictive Power of LV Ejection Fraction

LV ejection fraction was available in 689 patients, with a mean value of 64 ± 15%. In 69 patients LV ejection fraction was < 45% (range 23%-44%). LV ejection fraction was significantly associated with death at univariable (hazard ratio 0.96; 95% CI 0.94-0.98, *P* < .001) but not at multivariable analysis including age, gender, diabetes, METS, SDS ≥ 5%, and HRR (adjusted hazard ratio 0.98; 95% CI 0.96-1.01, *P* = .21).

## Discussion

To the best of our knowledge, this is the first study investigating the prognostic role of chronotropic incompetence assessed by HRR in a cohort of patients undergoing physical exercise stress-MPS for suspected CAD. Indeed, previous studies performed to assess if HRR adds incremental prognostic value to MPS included in the analysis both patients with and without known CAD, and the follow-up was shorter than in the present investigation.^19^,^20^ The main finding of the study is that after a follow-up of at least 5 years, HRR had independent and incremental prognostic value in a multivariable model including demographic data and clinical characteristic as well the occurrence of stress-induced myocardial ischemia at MPS. The prognostic role of clinical risk factors and stress-MPS in prediction of adverse outcome has been largely demonstrated.^21^–^23^ The results observed in the present study are in agreement with those investigating the role of HRR in patients undergoing exercise stress test.^5^,^24^ In particular, Cheng et al^24^ explored the relationship between HRR and all-cause mortality in healthy men and found that HRR was a strong predictor of cardiovascular mortality. Our findings extend Cheng results to both genders population characterized by co-morbidities and/or cardiac risk factors, highlighting the incremental prognostic value of this parameter in a model including, over clinical data also imaging findings.

The overall prognostic usefulness of measuring HR dynamic changes with exercise has been explored in a number of studies.^5^,^25^–^27^ In particular, Kubrychtova et al^25^ demonstrated that HR recovery after exercise may have a role in outcome prediction of patients with heart failure. The measure of HR recovery after exercise expresses autonomic system integrity which could be impaired not only in patients with heart failure but also in patients with stable CAD.^27^ The relationship of cardiac autonomic activation and HR response to pharmacological stress test has been also assessed in a population of patients with suspected or known CAD undergoing stress-MPS and ^123^I-*meta*iodobenzylguanidine imaging proposing that chronotropic response to stress test may be considerate a surrogate marker of sympathetic impairment.^27^ HR response to pharmacological stress-imaging was also assessed by Cortigiani et al^28^ who showed, in a large cohort of 3,059 patients undergoing high-dose dipyridamole stress echocardiography, that blunted HRR is a useful non-imaging prognostic parameter. In a different study from the same group,^29^ the prognostic value of HR response to pharmacological stress was also demonstrated in patients with permanent atrial fibrillation. Hage et al^30^ found that HRR to regadenoson in the lowest quartile independently predicted mortality after 22 months of follow-up in patients undergoing stress MPS. Moreover, a blunted HRR to adenosine had incremental value to stress MPS in a cohort of high-risk patients with diabetes mellitus and chronic kidney disease.^31^ Finally, in agreement with our results, Gebhard et al^8^ found that blunted HRR to adenosine stress was of incremental prognostic value in women over CAD risk factors and imaging findings.

As peak/rest HR ratio has been reported to be a significant predictor of events,^15^ we evaluated its prognostic value in our study population. When peak/rest HR ratio and HRR were included in the same multivariable model, HRR was significant independent predictor, but peak/rest HR ratio was not.

Yet, data reported in the present investigation highlight the importance to take into account the chronotropic responsiveness to stress not only in frail patients who need to undergo pharmacological tests due to poor exercise tolerance, but also in those with such a level of exercise capacity as to allow physical stress-MPS. Our findings also demonstrate the incremental prognostic value of HRR over clinical data and stress-MPS results.

Nevertheless, some limitations should be considered. This is a single center experience regarding only patients with suspected CAD. Due to the retrospective nature of the study, the cause of death could not be reliably defined for many patients. Therefore, only all-cause mortality was considered as an outcome, and it was not possible to analyze whether and how much of the excess mortality associated with the reduced HRR was related to cardiovascular mortality. Noteworthy, in the study of Azarbal and colleagues^20^ chronotropic incompetence remained predictive for death also after correction for CAD. Thus, chronotropic incompetence may imply the presence of autonomic dysfunction, which relates to mortality.

Finally, data concerning biomarkers were not available. Further investigations may consider the possible value of chronotropic responsiveness biomarkers in addition to stress-MPS variables in the outcome prediction.

## New Knowledge Gained

Our study adds new information about the long-term incremental prognostic role of HRR in patients with suspected CAD undergoing exercise stress-MPS. Chronotropic responsiveness capacity after stress-MPS should be considered not only in frail patients requiring pharmacological stress but also in those performing physical workout.

## Conclusion

Chronotropic incompetence assessed by HRR evaluation, has independent and incremental prognostic value in predicting all-cause mortality in patients with suspected CAD undergoing exercise stress-MPS. Thus, the evaluation of such a parameter may further improve patients’ risk stratification during imaging tests.

## Supplementary Information

Below is the link to the electronic supplementary material.

Supplementary file1 (PPTX 246 kb)

Supplementary file2 (AAC 7833 kb)

## Abbreviations

MPS: Myocardial perfusion single-photon emission computed tomography
HR: Heart rate
HRR: Heart rate reserve
CAD: Coronary artery disease
ECG: Electrocardiography
SDS: Summed difference score
CI: Confidence interval
LV: Left ventricular
METS: Metabolic equivalents
