# Supplementary material for: Prognostic value of heart rate reserve in patients with suspected coronary artery disease undergoing stress myocardial perfusion imaging
Source: J Nucl Cardiol. 2021 Aug 3;29(5):2521–30. doi: 10.1007/s12350-021-02743-2 (PMC9553802; doi:10.1007/s12350-021-02743-2)
Supplement: Supplementary file 1 — Supplementary file1 (PPTX 246 kb) [file 12350_2021_2743_MOESM1_ESM.pptx]

## Slide 1
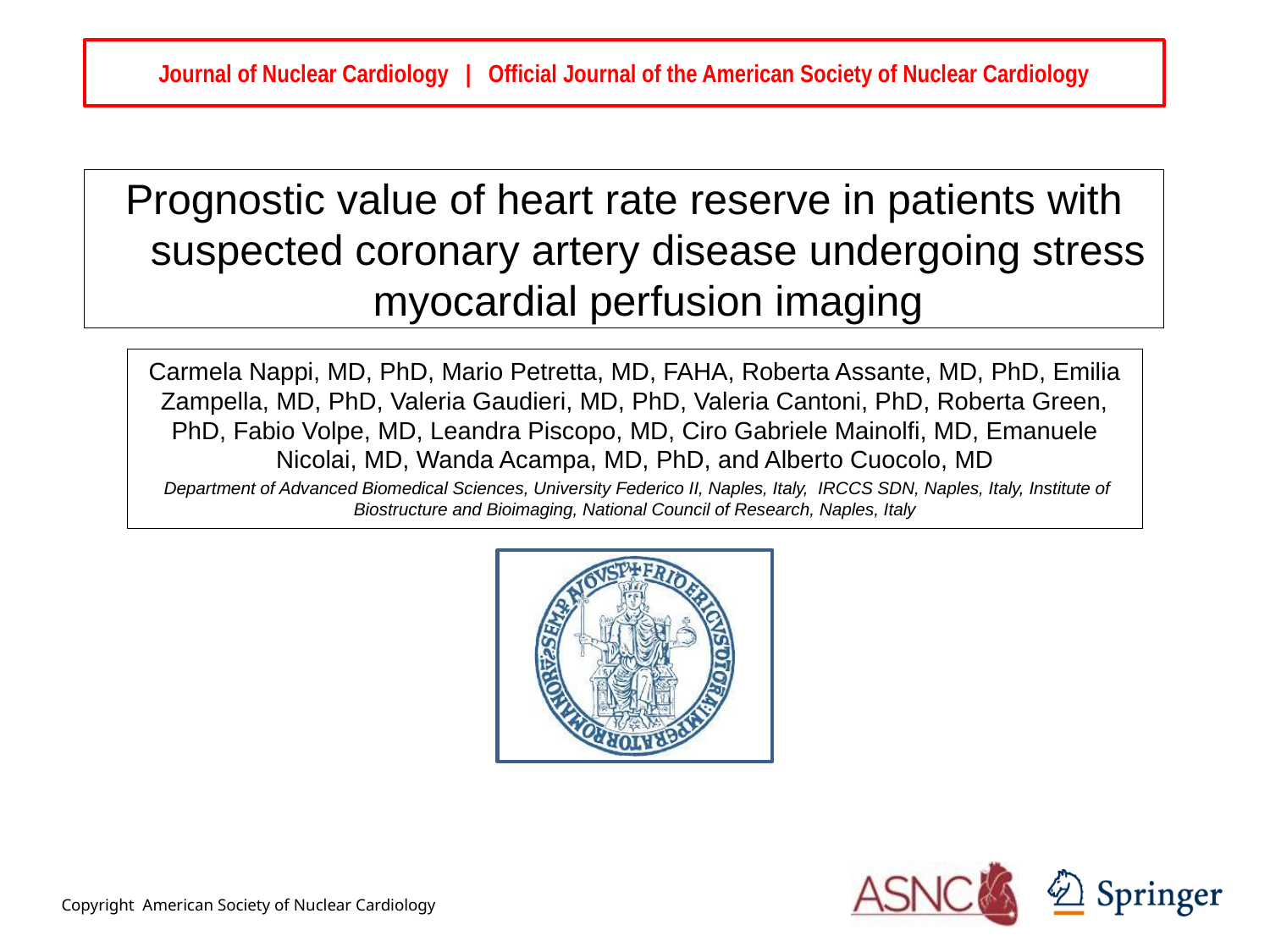

Journal of Nuclear Cardiology | Official Journal of the American Society of Nuclear Cardiology
# Prognostic value of heart rate reserve in patients with suspected coronary artery disease undergoing stress myocardial perfusion imaging
Carmela Nappi, MD, PhD, Mario Petretta, MD, FAHA, Roberta Assante, MD, PhD, Emilia Zampella, MD, PhD, Valeria Gaudieri, MD, PhD, Valeria Cantoni, PhD, Roberta Green, PhD, Fabio Volpe, MD, Leandra Piscopo, MD, Ciro Gabriele Mainolfi, MD, Emanuele Nicolai, MD, Wanda Acampa, MD, PhD, and Alberto Cuocolo, MD
 Department of Advanced Biomedical Sciences, University Federico II, Naples, Italy, IRCCS SDN, Naples, Italy, Institute of Biostructure and Bioimaging, National Council of Research, Naples, Italy
Copyright American Society of Nuclear Cardiology

## Slide 2
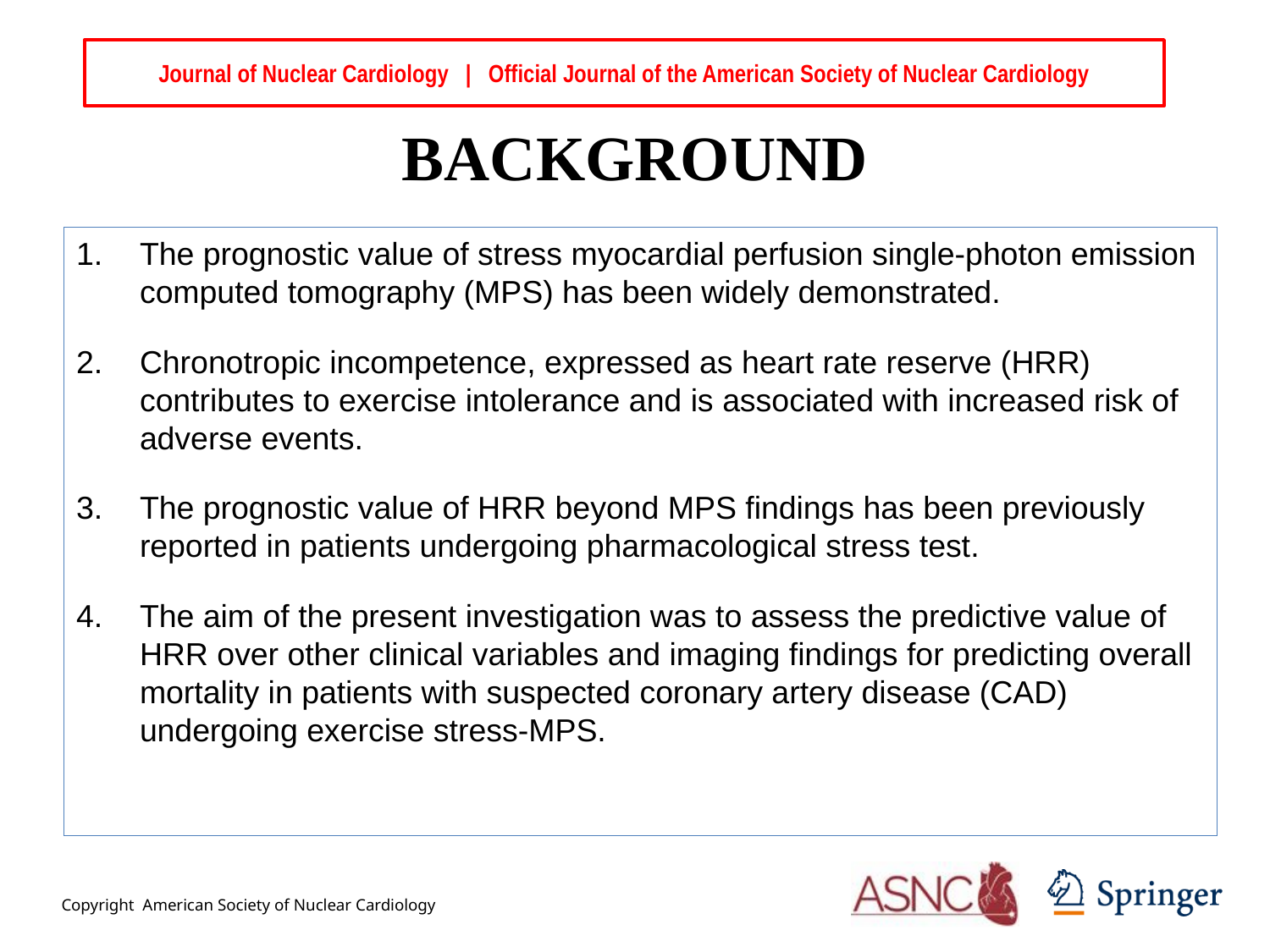

Journal of Nuclear Cardiology | Official Journal of the American Society of Nuclear Cardiology
# BACKGROUND
The prognostic value of stress myocardial perfusion single-photon emission computed tomography (MPS) has been widely demonstrated.
Chronotropic incompetence, expressed as heart rate reserve (HRR) contributes to exercise intolerance and is associated with increased risk of adverse events.
The prognostic value of HRR beyond MPS findings has been previously reported in patients undergoing pharmacological stress test.
The aim of the present investigation was to assess the predictive value of HRR over other clinical variables and imaging findings for predicting overall mortality in patients with suspected coronary artery disease (CAD) undergoing exercise stress-MPS.
Copyright American Society of Nuclear Cardiology

## Slide 3
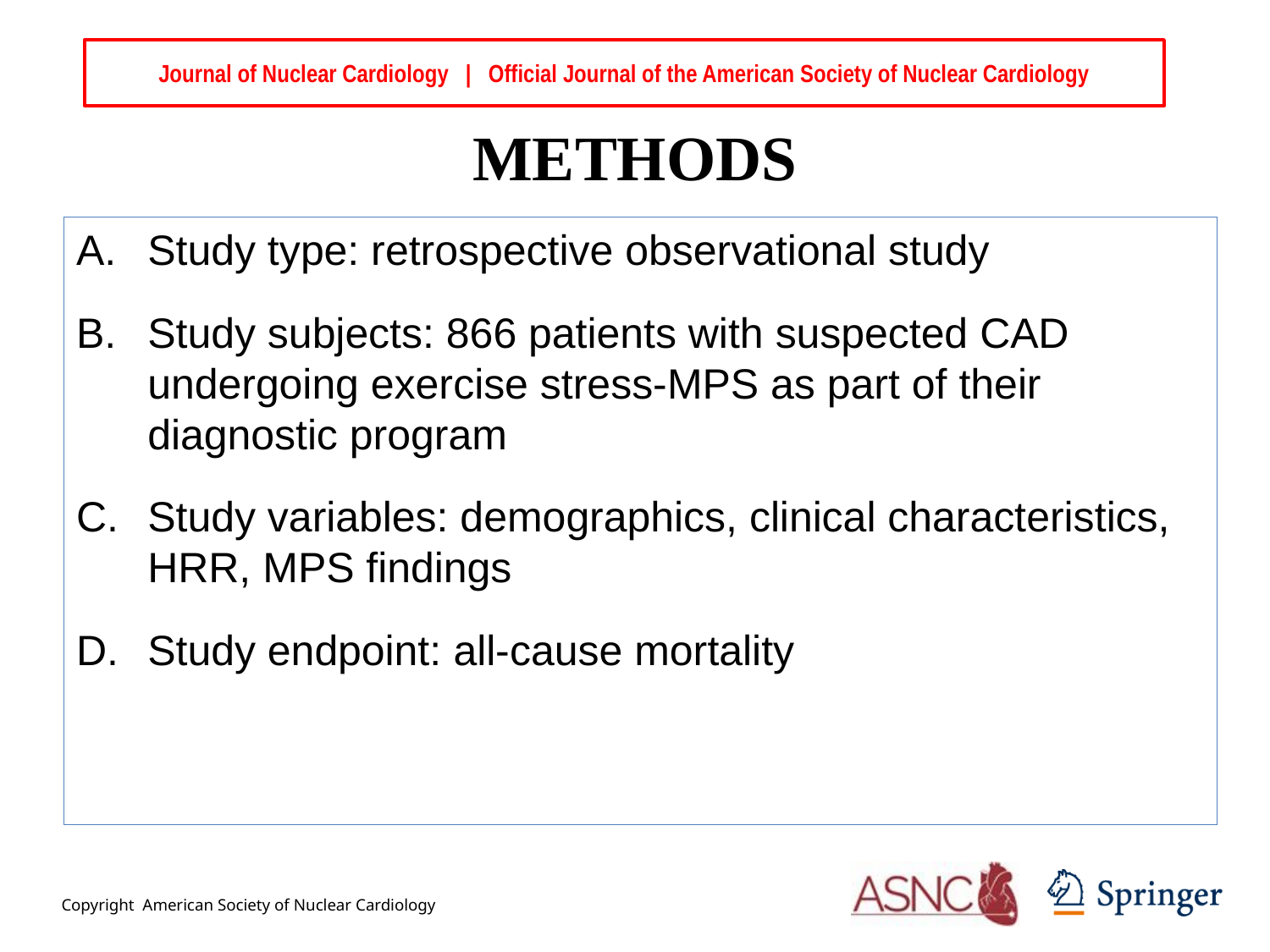

Journal of Nuclear Cardiology | Official Journal of the American Society of Nuclear Cardiology
# METHODS
Study type: retrospective observational study
Study subjects: 866 patients with suspected CAD undergoing exercise stress-MPS as part of their diagnostic program
Study variables: demographics, clinical characteristics, HRR, MPS findings
Study endpoint: all-cause mortality
Copyright American Society of Nuclear Cardiology

## Slide 4
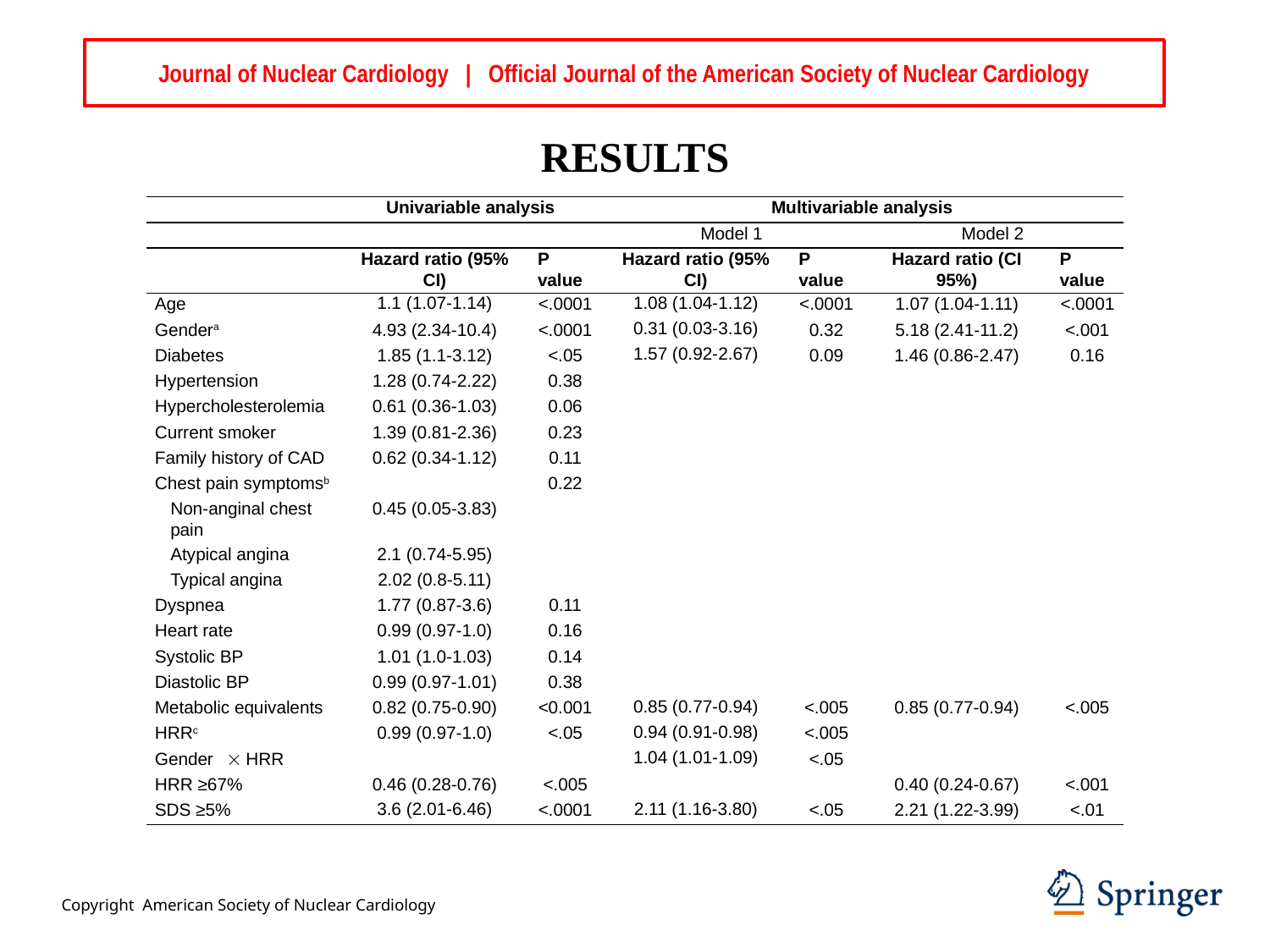

Journal of Nuclear Cardiology | Official Journal of the American Society of Nuclear Cardiology
RESULTS
| | Univariable analysis | | Multivariable analysis | | | |
| --- | --- | --- | --- | --- | --- | --- |
| | | | Model 1 | | Model 2 | |
| | Hazard ratio (95% CI) | P value | Hazard ratio (95% CI) | P value | Hazard ratio (CI 95%) | P value |
| Age | 1.1 (1.07-1.14) | <.0001 | 1.08 (1.04-1.12) | <.0001 | 1.07 (1.04-1.11) | <.0001 |
| Gendera | 4.93 (2.34-10.4) | <.0001 | 0.31 (0.03-3.16) | 0.32 | 5.18 (2.41-11.2) | <.001 |
| Diabetes | 1.85 (1.1-3.12) | <.05 | 1.57 (0.92-2.67) | 0.09 | 1.46 (0.86-2.47) | 0.16 |
| Hypertension | 1.28 (0.74-2.22) | 0.38 | | | | |
| Hypercholesterolemia | 0.61 (0.36-1.03) | 0.06 | | | | |
| Current smoker | 1.39 (0.81-2.36) | 0.23 | | | | |
| Family history of CAD | 0.62 (0.34-1.12) | 0.11 | | | | |
| Chest pain symptomsb | | 0.22 | | | | |
| Non-anginal chest pain | 0.45 (0.05-3.83) | | | | | |
| Atypical angina | 2.1 (0.74-5.95) | | | | | |
| Typical angina | 2.02 (0.8-5.11) | | | | | |
| Dyspnea | 1.77 (0.87-3.6) | 0.11 | | | | |
| Heart rate | 0.99 (0.97-1.0) | 0.16 | | | | |
| Systolic BP | 1.01 (1.0-1.03) | 0.14 | | | | |
| Diastolic BP | 0.99 (0.97-1.01) | 0.38 | | | | |
| Metabolic equivalents | 0.82 (0.75-0.90) | <0.001 | 0.85 (0.77-0.94) | <.005 | 0.85 (0.77-0.94) | <.005 |
| HRRc | 0.99 (0.97-1.0) | <.05 | 0.94 (0.91-0.98) | <.005 | | |
| Gender × HRR | | | 1.04 (1.01-1.09) | <.05 | | |
| HRR ≥67% | 0.46 (0.28-0.76) | <.005 | | | 0.40 (0.24-0.67) | <.001 |
| SDS ≥5% | 3.6 (2.01-6.46) | <.0001 | 2.11 (1.16-3.80) | <.05 | 2.21 (1.22-3.99) | <.01 |
Copyright American Society of Nuclear Cardiology

## Slide 5
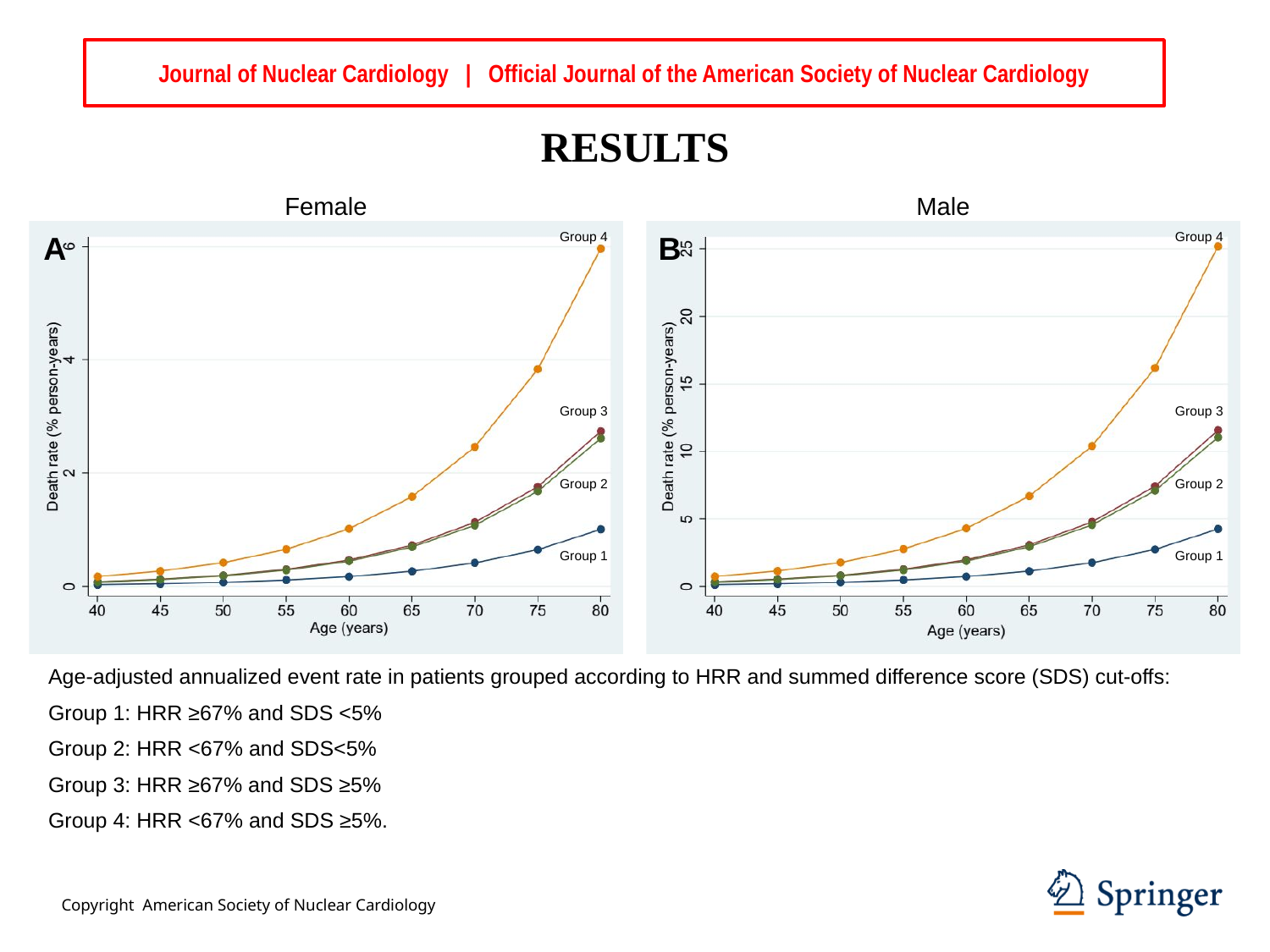

Journal of Nuclear Cardiology | Official Journal of the American Society of Nuclear Cardiology
RESULTS
# Female
Male
A
Group 4
Group 3
Group 2
Group 1
B
Group 4
Group 3
Group 2
Group 1
Age-adjusted annualized event rate in patients grouped according to HRR and summed difference score (SDS) cut-offs:
Group 1: HRR ≥67% and SDS <5%
Group 2: HRR <67% and SDS<5%
Group 3: HRR ≥67% and SDS ≥5%
Group 4: HRR <67% and SDS ≥5%.
Copyright American Society of Nuclear Cardiology

## Slide 6
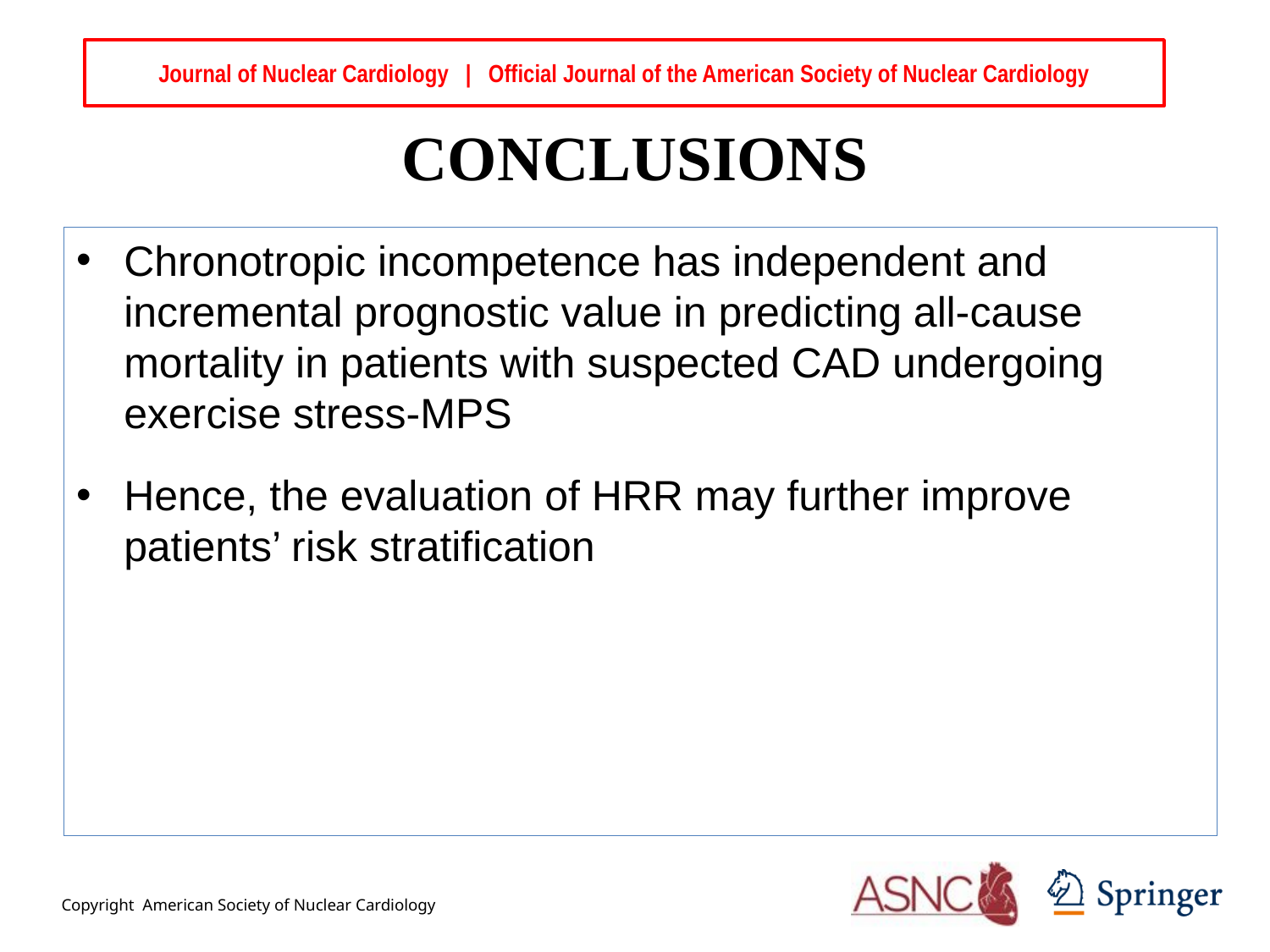

Journal of Nuclear Cardiology | Official Journal of the American Society of Nuclear Cardiology
# CONCLUSIONS
Chronotropic incompetence has independent and incremental prognostic value in predicting all-cause mortality in patients with suspected CAD undergoing exercise stress-MPS
Hence, the evaluation of HRR may further improve patients’ risk stratification
Copyright American Society of Nuclear Cardiology
